# Patterns of Interdependence between Financial Development, Fiscal Instruments, and Environmental Degradation in Developed and Converging EU Countries

**DOI:** 10.3390/ijerph17124425

**Published:** 2020-06-19

**Authors:** Magdalena Zioło, Krzysztof Kluza, Jarosław Kozuba, Miroslav Kelemen, Piotr Niedzielski, Paweł Zinczak

**Affiliations:** 1 Department of Sustainable Finance and Capital Markets, University of Szczecin, 71-101 Szczecin, Poland; magdalena.ziolo@usz.edu.pl (M.Z.); pzinczak@gmail.com (P.Z.); 2 Department of Quantitative Economics, Warsaw School of Economics, 02-554 Warsaw, Poland; kkluza@sgh.waw.pl; 3 Faculty of Transport, Silesian University of Technology, 44-100 Gliwice, Poland; 4 Faculty of Aeronautics, Technical University of Kosice, 04-121 Kosice, Slovakia; miroslav.kelemen@tuke.sk; 5 Department of Enterprise Management, University of Szczecin, 71-007 Szczecin, Poland; piotr.niedzielski@usz.edu.pl

**Keywords:** sustainability, finance, greenhouse gas emission, government, policy

## Abstract

Environmental risks, in particular climate change and environmental pollution, are among the key challenges faced by modern governments nowadays. Environmental risks are associated with specific costs and expenditures necessary to mitigate their negative effects. In this context, the financial system plays a significant role, particularly the public financial system, which allocates and redistributes public resources and has an impact on market participants by imposing environmental taxes. This study assessed the interdependence between environmental degradation and public expenditure, financial sector development, environmental taxes, and related socioeconomic policies. The aim was to diagnose and define the relationship between environmental degradation and sustainable fiscal instruments used in the financial system. The original research approach adopted in the study is the inclusion of variables representing a sustainable approach to assessment of the financial system. Two groups of European Union countries were analyzed for the period 2008–2017, namely, converging economies from Central and Eastern Europe and the largest developed economies of Western Europe. The authors found a strong relationship between greenhouse gas emissions and fiscal instruments, especially expenditure on research and development, and the development of the financial sector. In the case of environmental taxes, their impact differed depending on the country, being predominantly beneficial in countries with higher greenhouse gas emissions but unfavorable in countries with lower emissions levels.

## 1. Introduction

Ongoing socioeconomic and environmental changes, including the growing role of nonfinancial factors as risk factors (environmental, social, and governance (ESG) factors), make them crucial in financial management, particularly for risk management processes at the state level and in financial market institutions [1]. The growing role of environmental risks [2] has prompted the need for new measures in order to skillfully mitigate that type of risk. In response, new subdisciplines of finance have developed, such as carbon finance, climate finance, green finance, or a combination of all these environmental finance categories.

The finance paradigm. In the scope of sustainable finance, significant emphasis is conventional finance paradigm is gradually being replaced by the sustainable especially placed on providing financing for low-carbon technologies and influencing pro-environmental decisions of market participants by imposing tax and expenditure instruments. A special role in this respect is attributed to environmental taxes, including carbon taxes and research and development (R&D) expenditures, which support innovative pro-environmental solutions. However, we should remember that both environmental tax and environmental expenditure policies are conducted independently by individual member states of the European Union (EU). Hence, the fiscal effectiveness of individual expenditure and tax instruments and their impact on environmental performance remains varied between European Union countries. Differences between countries result from not only the lack of a common environmental tax policy but also the different positions of individual countries owing to the level of their greenhouse gas emissions, the sectoral structure of the economy, energy productivity, or the type of state and its development level (developed country, converging economy, etc.).

This study aimed to identify and define the relationship between environmental degradation and sustainable fiscal instruments used in the financial system. For the purpose of the study, the research hypotheses were as follows: (1) the more sustainable the public financial system, the stronger the impact of fiscal instruments on environmental degradation and the greater the impact on the level of greenhouse gas emissions; (2) the higher the degree of leverage, the less sustainable the financial system. In other words, the more sustainable the public financial system, the more developed the environmental taxation system will be and the higher the share of expenditure on environmental protection will be in relation to gross domestic product (GDP). The more sustainable the commercial financial system, the lower the degree of financial leverage will be. The specific objectives of the study were as follows [3]:

- diagnosing differences between countries with regard to greenhouse gas emissions and sustainable financial systems and
- assessing the impact of environmental tax and expenditures within the sustainable public financial system and defining in which countries these instruments are the most effective.

The authors found a strong link between greenhouse gas emissions and fiscal instruments, particularly the expenditure on research and development, and the development of the financial sector. Two groups of European Union countries were analyzed for the period 2008–2017, namely, converging economies from Central and Eastern Europe (Bulgaria, Czech Republic, Hungary, Poland, Romania, and Slovakia) and the largest developed economies of Western Europe (Germany, Spain, France, UK, Netherlands, and Italy). The impact of environmental taxes varied from country to country, being particularly advantageous in countries with higher greenhouse gas emissions and unfavorable in countries with lower emission levels [4]. In the developed economies of Western Europe, this impact was higher than in converging EU countries and the EU average.

The rest of the paper is organized as follows. In Section 2, the research problem is presented and related work is discussed in the context of defining the research gap. Section 3 presents the materials, variables, and a description of the research methods. The main research results and discussion are presented in detail in Section 4. Finally, Section 5 presents the conclusions and the original contribution of this work to the existing literature on this subject matter.

## 2. Theoretical Framework and Related Papers

Do factors such as gasoline combustion have an impact on the increased amount of greenhouse gases in the atmosphere? In their article, Richard Wood and Edgar Hertwich pointed to this element as a natural factor increasing emissions in Europe [5]. They also stated, on the basis of statistical surveys, that this factor has no tendency to decrease but rather to increase. Other factors that influence the emission of gases are the mass production of meat and the development of cities or factories. David Dodman, quoting Sánchez-Rodríguez, pointed out that the diversity of impacts can be grouped into two broad categories: those originating in urban areas that have a negative effect on the global environmental change and global environmental changes that exert negative effects on urban areas [6]. He indicated that cities are not the largest source of pollution. He also claimed that greenhouse gas production per capita is definitely lower in cities than in any other place. Dodman also discussed a problem related to the type of city or “specialization” in a given area. An industrial city will obviously create more carbon dioxide and other pollution than a tourist city [6]. The large number of factors that affect environmental degradation creates a problem related to the choice of environmental protection. One of the possible solutions in this case seems to be the use of state aid, in particular fiscal measures that affect the everyday life of society [7].

Looking at the literature related to the impact of fiscal instruments on environmental degradation, many different approaches to this topic can be noticed. Jerzy Śleszyński (2014), in his article “Environmental Taxes and Division into Groups of Taxes According to the Eurostat Methodology” pointed out the problems with the appropriate definition of environmental taxes. Based on his approach, four tax groups are listed [8]:

- taxes on energy, which include fees for using energy carriers, such as natural gas, oils, gasoline, or diesel, as well as a fee for the emission of carbon dioxide;
- taxes on means of transport, which include taxes for the possession and use of transport vehicles and taxes related to transport services;
- taxes on air pollution, excluding carbon dioxide emissions; and
- taxes on natural resources, which include taxes on the use of natural resources and taxes on oil and gas.

The author analyzed the problems related to the proper categorization of environmental taxation, taking into account the four groups of taxes mentioned above. He also pointed out the characteristics of an environmental tax, among them [8], the characteristic that “environmental tax must have a harmful effect on the natural environment as a physical unit of negative impact or its specifically specified substitute”. Śleszyński argued that it is difficult to introduce appropriate tax benefits for people who display environmentally positive behavior [8]. In the European Environmental Economic Funds Program, there is a module for environmental taxes, and it is worth mentioning that no subsidies that can act as stimuli to protect the environment are taken into account in it. Śleszyński also stated that the division of taxes is varied only at the first glance because one can see how the taxes have shifted towards energy tax [8]. The use of gasoline or diesel are included as atmospheric pollutants, but CO_2_ emissions have been excluded [8].

R.K. Turner, R. Salmons, J. Powell, and A. Craighill in their 1998 paper discussed an ideal tax approach and argued that optimal environmental taxation should take into consideration adequate economic efficiency, which is aimed at shaping an appropriate price level while maintaining an appropriate reason and which should be effective in the context of environmental protection. The article pointed to the problem concerning appropriate tax formation and its effects while maintaining the experiences from previous events [9]. To sum up, imposing environmental tax should not adversely affect the budget of households, and the costs of taxation should be very low. Environmental tax should also be flexible and adaptable to the prevailing legal conditions in a given country and should be self-sufficient.

At the same time, the authors pointed out the problem of measuring negative effects [9]. In the article, UK landfill tax was given as a tax formula. This tax is aimed at increasing the cost of storage in order to provide a source of financing for activities aimed at caring for the environment [9]. The article “Green Taxes and Double Dividends in a Dynamic Economy” by Gerhard Glomm, Daiji Kawaguchi, and Facundo Sepulveda approached green taxes in two ways: positive and normative [10]. In the description of their ideal tax, the following elements were used: amount of fuel, health of the society at the time of use, and positive or negative effect on consumption. In the study presented in the article, it was concluded that the approach has positive effects, with the assumption that the price of fuel increases. The authors showed a downward trend in the use of cars by households [11]. The approach of such a tax to companies is more specific and is related to the size of the company as well as the amount of gasoline it uses. One of the main results of this project was the possibility of reducing existing taxes in favor of green taxes [8]. Philippe Thalman (2004) said that people who have stable employment are more likely and willing to agree to pay green taxes or other environmental charges [12]. He also pointed out that age plays a very important role in the approach to environmental protection. Young and well-educated people will not have any prejudices against environmental programs, fees, or taxes. Middle-aged people will be less willing to accept further fees, but here one can divide their attitudes depending on the level of education of a given person. The retired will be against any way of caring for the environment because for them, the long-term effects will not bring any tangible benefits and they will rather focus on their financial stability [13]. Another hypothesis put forward by this author was that there is greater willingness to pay such fees when they concern producers rather than consumers themselves. He also pointed out that the Swiss society has a completely different approach to the size of these taxes. They prefer many smaller fees that bring some financial exemptions rather than large and demanding taxes. Such a study also indicates the huge diversity among social groups, in particular those who own cars and those who do not. Swiss citizens support a green tax that will not be too intrusive on their home budgets. Dorota Burzyńska presented the problem of introducing taxes due to the level of economic development [14]. In highly developed countries, the introduced tax reform has caused high environmental benefits. In Sweden, the reduction of taxes related to energy income in agriculture and additional education as well as the imposition of tax on CO_2_ and SO_2_, reduced the use of poor quality fuels in agriculture. When Denmark introduced taxation on tap water, it reduced household water consumption. The author believed that the idea of carrying out reform is to transfer the burden from existing taxes to taxes on the use of natural resources. This will contribute to increasing social responsibility through the following [14]:

- continuous development of science aimed at reducing costs, which contributes to structural changes in the economy;
- change in the behavior of customers and producers, who will not think politically alone;
- reduction of environmental pollution; and
- internalization of external costs by taking into account the real costs of environment protection in the price of a given product or service.

In her approach, the author referred to the first tax reform introduced in England, where the British economist Arthur C. Pigou created a completely new and innovative approach to taxation, which can be considered as the basis of green tax [14]. This tax is based on correcting the market price of products that have a negative effect on society. She pointed out that ecological tax reform can contribute to economic and social benefits and raised the followed question: Does this approach allow a correct adjustment of expenses related to environmental protection? [14]

Anna Alberini and Kathleen Segerson (2002) described the approach to spending on environmental protection as an action of an individual that should be motivated by government programs, institutions related to environmental care, or companies that want to be competitive in the market [15]. They also pointed to the fact that environmental protection is a rising trend. In their research, they showed that 33 out of 50 respondents who were involved in improving environmental protection succeeded in their actions. They also confirmed the dependence of costs related to expenses on the environment with the types of taxation [15]. “Corporate Expenditure on Environmental Protection” by Stefanie A. Haller and Liam Murphy discussed the corporate approach to the problem of spending on environmental protection [16]. The authors pointed out the relationship between the size of a company and its willingness to implement elements related to environmental expenditure. When analyzing the Irish market, they noticed that foreign companies are much more likely to use environmental programs and try to create a nature-friendly brand than domestic, Irish companies [16].

## 3. Materials and Methods

In this article, the authors aimed to identify the relationship between environmental degradation and variables describing financial development as well as various socioeconomic policies of governments in European Union countries. Based on the literature review, greenhouse gas emissions were used as an explanatory variable representing environmental degradation [17]. Specifically, the authors used the emissions of greenhouse gases (CO_2_, N_2_O in CO_2_ equivalent, CH_4_ in CO_2_ equivalent) in kilograms per capita indicator from the Eurostat database.

Explanatory variables were divided into three groups distinguishing variables that represent financial sector development, related fiscal and socioeconomic conditions, and research and development activities by governments in the linked fields. The study was based on the selected indicators used to monitor the implementation of the objectives of the Agenda for Sustainable Development 2030 (Agenda 2030) [18,19]. They are presented in Table 1. The authors deliberately excluded from the analysis any variables more directly related to environmental characteristics of economies, such as “share of renewable energy in gross final energy consumption” and “energy productivity” [19]. Thus, our study was focused entirely on the effects that can be transmitted through social, economic, or financial channels.

The data was collected for the two groups of European Union countries. The first group of countries consisted of the largest EU converging economies from Central and Eastern Europe, i.e., the Visegrad Four (Poland (PL), the Czech Republic (CZ), Slovakia (SK), and Hungary (HU)), Bulgaria (BG), and Romania (RO). The second group comprised the largest Western European developed economies represented by Germany (DE), France (FR), the United Kingdom (UK), Italy (IT), Spain (ES), and the Netherlands (NL), which are also the largest emitters of greenhouse gases in the EU in nominal terms. All data was extracted from the Eurostat database for the period 2008–2017 to ensure full integrity and comparability of the data. The only missing data was for Poland, where values were omitted for the variables “government support to agricultural research and development” and “government support to environmental research and development” in the period 2009–2011.

The variables analyzed had very different denominations and value domains. Some were in nominal terms, while others were represented by per capita or % of GDP indicators. To overcome this issue, the authors applied a standardization procedure to all variables. As a result, the analysis conducted was based on a three-stage process:

1. standardization of variables based on standard N (0,10) distribution;
2. calculation of correlations between the dependent variable “greenhouse gas emissions” and all the explanatory variables; and
3. verifying the direction of relationships using a principal component analysis, i.e., analysis of component signs for the primary compound factor, which typically explained over 65% of the whole setup.

The results of Stage 2 are presented and discussed below in Section 4 of this article. The results of the principal component analysis (Stage 3) are shown in Appendix A.1, Appendix A.2, Appendix A.3, Appendix A.4, Appendix A.5, Appendix A.6, Appendix A.7, Appendix A.8, Appendix A.9, Appendix A.10, Appendix A.11 and Appendix A.12 (Table A1, Table A2, Table A3, Table A4, Table A5, Table A6, Table A7, Table A8, Table A9, Table A10, Table A11, Table A12, Table A13, Table A14, Table A15, Table A16, Table A17, Table A18, Table A19, Table A20, Table A21, Table A22, Table A23 and Table A24). It is worth emphasizing that the values for the first principal components (PC1) for respective variables confirmed, in general, the direction of the relationships from Stage 2.

## 4. Results and Discussion

The analysis undertaken revealed several meaningful relationships across the variables as well as across the groups of countries. Firstly, there was a remarkable difference in the impact exerted by the financial sector and public policies on environmental degradation in developed and converging economies. The interdependence of these variables was much stronger in the developed EU countries, which are the global leaders in environmental protection activities. The values of all correlation ratios are shown in Table 2.

For a better interpretable picture of dependencies across countries and variables, it was important to test the significance of particular correlation ratios. The results of such a procedure are shown in Table 3, which presents the probability of occurrence of zero correlation between the variable “greenhouse gas emissions” and all other variables. In general, values above 0.1 indicate statistically significant error in assuming that there was a nonzero correlation (i.e., with a probability above 10%). Values below 0.1 indicate that there was a statistically significant nonzero correlation between a given variable and the variable “greenhouse gas emissions”.

Analyzing the results from Table 2 and Table 3, it is worth noting that there was a strong relationship between governmental research and development policies and improved environmental characteristics of a given country, both in developed and converging EU economies. This was true for general research activities as well as, to a lesser extent, the ones related to environmental protection, indirectly proving the effectiveness of such public policies. To a lesser degree, there was a clear pattern with research on agriculture. Some significant exceptions were observed in Spain and Italy. This may reflect the different industrial structure of these economies (for example, the gross value coming from agriculture, forestry, and fishing industries in Spain and Italy amounted to 2%–3% of GDP in 2017–2018, while it amounted to 0.6–0.8% GDP in Germany and the UK). A similar situation takes place in Romania (with this ratio amounting to 4% of GDP).

Analysis of variables reflecting different public policies showed a remarkable correlation in several countries between improved environment state and increased government expenditure on environmental protection. Mixed results were obtained with indirect policies, such as government expenditure on education. Moreover, the analysis did not reveal a clear relationship between income inequalities and environmental degradation in the EU countries under consideration. Likewise, in the vast majority of countries, there was no correlation between the dependent variable and employment in high- and medium-high technology manufacturing sectors and knowledge-intensive service sectors. These observations support the case for using direct policy instruments with respect to curbing environmental degradation.

In addition to the above discussion, environmental taxes turned out to be an ambiguous policy instrument. In the group of developed countries, they tended to help the country’s environmental characteristics, with the remarkable exception of Germany, where they were explicitly inferior to other public policies and R&D activities. On the other hand, in converging EU economies, they tended to be counterproductive or negligible with respect to the limitation of greenhouse gas emissions [20].

Important conclusions may be drawn from this analysis with regard to variables representing development of the financial sector [21]. The analysis showed that development of the financial sector, in general, was strongly correlated with growing environmental degradation. This provides an additional argument for the need to develop sustainable financial products that incorporate environmental issues in business practice. This process should be facilitated in practice by government programs because the financial sector will not provide such solutions in an adequate number based on organic growth, as indicated by the data for developed EU countries. The analysis also showed that corporate bonds were the noteworthy exception from this conclusion. These are typically custom-tailored instruments, such as green bonds, which are suitable for tackling climate issues.

The graphical presentation of all the results is given in Table 4. The strength of the correlation was grouped in four typical categories. The correlation ratios with absolute values greater than 0.89 (which corresponds to 80% model fitness) were categorized as a “very strong relationship”, the ratios with absolute values between 0.63 and 0.89 (40% to 80% model fitness) were categorized as a “strong” relationship, and the ratios with absolute values between 0.45 and 0.63 (20% to 40% model fitness) were categorized as a “moderate relationship”. The remaining results (with absolute values below 0.45) were grouped in a “weak or lack of relationship” category. Table 4 makes it possible to easily identify the key similarities and differences between the largest EU converging economies from Central and Eastern Europe and the largest developed economies from Western Europe. In particular, increasing government expenditure on environmental protection and expenditure on R&D appeared to be effective policy instruments for reducing environmental degradation in EU countries, regardless of economic development levels of the country in the period 2008–2017.

## 5. Conclusions

This study identified the interdependencies between financial sector development as well as fiscal instruments dedicated to environmental protection and the level of greenhouse gas emissions. Preliminary results of analyses indicated the existing relationships between the variables studied. In the group of countries researched, strong positive relationships were observed between greenhouse gases and financial sector leverage and consolidating bank leverage. To sum up, the more developed the financial market and the greater the level of financial leverage, the higher was the volume of greenhouse gas emissions. The analysis of the public financial system in terms of fiscal instruments (expenditure and taxes affecting the environment) showed the reverse direction of the dependence. The higher the expenditure and environmental taxes, the lower was the level of greenhouse gas emissions. In particular, strong relationships between the variables were observed for developed countries, such as Germany, France, and Italy, that is, the countries with high greenhouse gas emissions (Germany is the leader). The research is preliminary and a contribution to further in-depth research. The original research approach adopted in the study is the inclusion of variables representing a sustainable approach to assessment of the financial system. Such an approach is novel. To the best of our knowledge, this is one of the first studies examining the relationship between financial and economic development, fiscal instruments, and environmental degradation. Studies published so far have focused on financial and economic development and environmental degradation but not on fiscal instruments (environmental taxes) reflecting a sustainable public finance component. The usual approach is therefore based on the assumption that the degree of sustainability of the public financial system determines environmental degradation. To sum up, the literature on the subject has focused on only analyzing the impact of financial and economic development on greenhouse gas emission while the fiscal component has been omitted in this context. The approach presented here is innovative also because the study includes sustainable public finance, while existing studies have been based only on the conventional finance approach. The research results presented so far show the relationship between greenhouse gas emissions and economic growth and development but do not take into account environmental and social variables in the analysis of that dependence. This study tried to expand the present approach and include variables referring to the environmental and social pillar of sustainable development. This approach allows one to check if sustainable finance is important for environmental degradation compared to the conventional approach.

## Figures and Tables

**Table 1:** Variables used in the research.

Field:	Variable:	Variable No.*
Environmental degradation (dependent variable)	Greenhouse gas emissions (CO_2_, N_2_O in CO_2_ equivalent, CH_4_ in CO_2_ equivalent), kilograms per capita	-
Financial sector development	Financial sector leverage (debt to equity), nonconsolidated, in %	1
	Consolidated banking leverage, domestic and foreign entities (asset-to-equity multiple)	2
	Private sector debt securities, nonconsolidated as % of GDP	3
	Private sector debt loans, nonconsolidated as % of GDP	4
Socioeconomic policies	Share of environmental taxes in total tax revenues GDP	5
	Employment in high- and medium-high technology CH_4_ manufacturing sectors and knowledge-intensive service sectors as % of total employment	6
	Inequality of income distribution (income quintile share ratio)	7
	General government expenditure on environmental protection (COFOG), million EUR	8
	General government expenditure on education (COFOG), million EUR	9
Research & development	Gross domestic expenditure on research and development as % of GDP	10
	Government support to agricultural research and development (GBOARD), Euro per inhabitant	11
	Government support to environmental research and development (GBOARD), Euro per inhabitant	12

* These variable numbers are henceforth used in Table 2, Table 3 and Table 4 to refer to the specific variables instead of using full variable names. Source: own analysis. GDP, gross domestic product; COFOG, Classification of the Functions of Government; GBOARD, government budget appropriations or outlays on research and development.

**Table 2:** Correlation ratios between the variable “greenhouse gas emissions” and explanatory variables; all variables were standardized.

Variable No.:	1	2	3	4	5	6	7	8	9	10	11	12
FR	0.32	0.91	−0.99	−0.85	−0.79	−0.75	0.93	−0.94	−0.92	−0.95	−0.73	0.50
DE	0.67	0.73	−0.62	0.37	0.56	−0.22	0.07	−0.70	−0.89	−0.66	−0.73	−0.64
IT	0.24	0.11	−0.97	0.70	−0.92	−0.41	−0.85	−0.97	−0.97	0.83	0.93	0.96
NL	0.85	0.75	−0.44	−0.81	0.77	0.02	0.30	−0.87	0.35	−0.55	0.65	−0.73
ES	0.37	0.68	−0.59	0.50	−0.60	0.06	−0.78	0.57	0.66	0.56	0.86	0.81
UK	0.88	0.97	−0.33	0.90	−0.31	0.73	0.31	−0.44	−0.26	−0.16	−0.74	0.90
BG	−0.02	0.32	−0.37	−0.18	0.42	0.01	−0.01	−0.30	−0.62	−0.27	−0.04	0.15
CZ	0.82	0.78	−0.83	−0.71	0.84	−0.77	−0.11	−0.91	−0.15	−0.60	0.50	0.38
HU	0.19	0.71	−0.58	0.12	0.32	−0.03	−0.60	−0.80	−0.44	0.54	0.10	0.21
PL	0.52	0.55	−0.46	−0.25	0.01	0.11	0.07	−0.38	−0.13	0.08	−0.56	−0.37
RO	0.91	0.60	0.26	0.46	−0.78	−0.40	−0.20	0.63	−0.51	0.52	0.65	0.03
SK	0.90	0.88	−0.77	−0.38	0.94	−0.40	−0.21	−0.85	−0.56	−0.94	0.35	−0.63

Note 1: A full description of the variables (and corresponding variable numbers) is presented in Table 1. Note 2: The results for variable no. 11 and no. 12 for Poland are based on unbalanced samples. Full data sets are available per request. Source: own analysis. FR, France; DE, Germany; IT, Italy; NL, the Netherlands; ES, Spain; UK, United Kingdom; BG, Bulgaria; CZ, Czech Republic; HU, Hungary; PL, Poland; RO, Romania; SK, Slovakia.

**Table 3:** Statistical significance of the correlation ratios from Table 2 (*p*-values) between the variable “greenhouse gas emissions” and explanatory variables.

Variable no.:	1	2	3	4	5	6	7	8	9	10	11	12
FR	0.36	**0.00**	**0.00**	**0.00**	**0.01**	**0.01**	**0.00**	**0.00**	**0.00**	**0.00**	**0.02**	0.14
DE	**0.03**	**0.02**	**0.06**	0.30	**0.09**	0.54	0.85	**0.00**	**0.04**	**0.02**	**0.02**	**0.05**
IT	0.50	0.76	**0.00**	**0.02**	**0.00**	0.24	**0.00**	**0.00**	**0.00**	**0.00**	**0.00**	**0.00**
NL	**0.00**	**0.01**	0.21	**0.00**	**0.01**	0.96	0.40	0.32	**0.10**	**0.00**	**0.04**	**0.02**
ES	0.29	**0.03**	**0.07**	0.14	**0.07**	0.88	**0.01**	**0.04**	**0.09**	**0.09**	**0.00**	**0.00**
UK	**0.00**	**0.00**	0.35	**0.00**	0.39	**0.02**	0.39	0.47	0.65	0.20	**0.01**	**0.00**
BG	0.97	0.36	0.29	0.62	0.23	0.98	0.98	**0.06**	0.45	0.40	0.91	0.68
CZ	**0.00**	**0.01**	**0.00**	**0.02**	**0.00**	**0.01**	0.77	0.68	**0.07**	**0.00**	0.14	0.28
HU	0.60	**0.02**	**0.08**	0.74	0.37	0.93	**0.06**	0.20	0.11	**0.01**	0.78	0.56
PL	0.12	**0.10**	0.18	0.49	0.97	0.77	0.86	0.72	0.82	0.29	*0.19*	*0.41*
RO	**0.00**	**0.06**	0.46	0.18	**0.01**	0.26	0.59	0.13	0.13	**0.05**	**0.04**	0.94
SK	**0.00**	**0.00**	**0.01**	0.28	**0.00**	0.25	0.56	**0.09**	**0.00**	**0.00**	0.32	**0.05**

Note 1: A full description of the variables (and corresponding variable numbers) is presented in Table 1. Note 2: *p*-values are calculated for a two-tailed critical area. Degrees of freedom = 8, with the exception of Poland for the variable no. 11 and no. 12 (5 degrees of freedom). Note 3: Significant correlations (*p*-values below 0.1) are indicated in bold. Source: own analysis.

**Table 4:** Characteristics of correlations between the variable “greenhouse gas emissions” and explanatory variables; all variables were standardized.

	Financial Sector Development				Socioeconomic Policies					Research and Development		
Variable no.:	1	2	3	4	5	6	7	8	9	10	11	12
**FR**												
**DE**												
**IT**												
**NL**												
**ES**												
**UK**												
**BG**												
**CZ**												
**HU**												
**PL**												
**RO**												
**SK**												

Legend (strength of correlation): dark green—very strong positive; green—strong positive; light green—moderate positive; dark red—very strong negative; red—strong negative; pink—moderate negative. A full description of variables (and corresponding variable numbers) is presented in Table 1. Note 2: Blank fields represent weak or zero correlations. Source: own analysis.

## Appendix A

Principal Factor Analysis (PCA) for the analyzed countries (Appendix A.1, Appendix A.2, Appendix A.3, Appendix A.4, Appendix A.5, Appendix A.6, Appendix A.7, Appendix A.8, Appendix A.9, Appendix A.10, Appendix A.11 and Appendix A.12). The first four principal components are presented (the number of significant eigenvalues is no greater than 4 for each analyzed country).

### Appendix A.1. (Germany)

**Table A1:** Eigenanalysis of the correlation matrix.

Component	Eigenvalue	Proportion	Cumulative
1	8.7379	0.6721	0.6721
2	1.5133	0.1164	0.7886
3	1.2464	0.0959	0.8844
4	0.6578	0.0506	0.9350

**Table A2:** Eigenvectors (component loadings).

	PC 1	PC 2	PC 3	PC 4
Variable 1	0.325	−0.120	−0.043	0.091
Variable 2	0.332	−0.082	0.050	0.067
Variable 3	−0.204	−0.464	0.044	−0.576
Variable 4	0.263	−0.323	−0.167	−0.420
Variable 5	0.303	0.085	−0.251	0.107
Variable 6	0.058	−0.665	0.215	0.583
Variable7	−0.025	0.032	0.852	−0.090
Variable 8	−0.310	−0.181	−0.180	−0.121
Variable 9	−0.333	0.106	0.037	0.065
Variable 10	−0.325	0.072	−0.092	0.157
Variable 11	−0.303	0.037	−0.192	0.238
Variable 12	−0.326	0.054	0.133	−0.044
GGE	0.258	0.394	0.183	−0.133

Note 1: GGE, “greenhouse gas emissions” (dependent variable). PC, principal component. Source: own analysis.

### Appendix A.2. (Spain)

**Table A3:** Eigenanalysis of the correlation matrix.

Component	Eigenvalue	Proportion	Cumulative
**1**	8.6516	0.6655	0.6655
**2**	2.3433	0.1803	0.8458
**3**	0.9562	0.0736	0.9193
**4**	0.6457	0.0497	0.9690

**Table A4:** Eigenvectors (component loadings).

	PC 1	PC 2	PC 3	PC 4
Variable 1	0.322	−0.116	−0.195	−0.101
Variable 2	−0.241	0.154	−0.479	−0.565
Variable 3	−0.179	0.420	0.492	−0.179
Variable 4	−0.289	−0.175	−0.087	0.362
Variable 5	0.245	−0.417	0.204	0.101
Variable 6	0.311	−0.202	0.253	0.029
Variable7	−0.310	0.190	0.102	0.261
Variable 8	0.306	−0.245	−0.142	−0.184
Variable 9	0.302	0.283	−0.021	−0.124
Variable 10	0.305	0.250	−0.125	−0.148
Variable 11	0.268	0.322	−0.206	0.331
Variable 12	0.228	0.368	−0.277	0.471
GGE	0.261	0.253	0.460	−0.159

Note 1: GGE, “greenhouse gas emissions” (dependent variable). Source: own analysis.

### Appendix A.3. (France)

**Table A5:** Eigenanalysis of the correlation matrix.

Component	Eigenvalue	Proportion	Cumulative
**1**	9.3471	0.7190	0.7190
**2**	1.3332	0.1026	0.8216
**3**	1.1802	0.0908	0.9123
**4**	0.6594	0.0507	0.9631

**Table A6:** Eigenvectors (component loadings).

	PC 1	PC 2	PC 3	PC 4
Variable 1	−0.310	−0.024	0.245	−0.072
Variable 2	−0.241	−0.152	−0.549	−0.008
Variable 3	−0.272	0.364	0.278	−0.069
Variable 4	0.299	0.209	0.249	−0.066
Variable 5	0.137	−0.668	0.292	0.401
Variable 6	0.310	−0.094	0.177	0.126
Variable7	−0.323	−0.050	−0.038	−0.021
Variable 8	−0.287	0.186	0.339	0.075
Variable 9	−0.252	0.048	0.280	0.569
Variable 10	0.157	0.485	−0.341	0.665
Variable 11	−0.292	−0.239	−0.251	0.190
Variable 12	−0.322	0.049	0.084	−0.009
GGE	0.322	0.102	0.038	−0.032

Note 1: GGE, “greenhouse gas emissions” (dependent variable). Source: own analysis.

### Appendix A.4. (UK)

**Table A7:** Eigenanalysis of the correlation matrix.

Component	Eigenvalue	Proportion	Cumulative
**1**	7.1289	0.5484	0.5484
**2**	2.5683	0.1976	0.7459
**3**	1.3586	0.1045	0.8504
**4**	1.1997	0.0923	0.9427

**Table A8:** Eigenvectors (component loadings).

	PC 1	PC 2	PC 3	PC 4
Variable 1	−0.148	−0.200	−0.470	−0.548
Variable 2	−0.253	0.129	0.296	−0.541
Variable 3	0.341	−0.082	0.064	0.304
Variable 4	0.212	−0.348	−0.387	−0.049
Variable 5	0.365	−0.001	0.058	0.071
Variable 6	0.360	0.055	0.099	−0.171
Variable7	−0.218	0.460	0.082	−0.060
Variable 8	0.351	0.110	−0.021	−0.129
Variable 9	−0.321	0.140	0.185	0.296
Variable 10	0.287	0.186	0.278	−0.303
Variable 11	−0.122	−0.525	0.332	−0.154
Variable 12	−0.080	−0.498	0.470	0.047
GGE	0.336	0.104	0.273	−0.227

Note 1: GGE, “greenhouse gas emissions” (dependent variable). Source: own analysis.

### Appendix A.5. (The Netherlands)

**Table A9:** Eigenanalysis of the correlation matrix.

Component	Eigenvalue	Proportion	Cumulative
1	7.8314	0.6024	0.6024
2	2.1602	0.1662	0.7686
3	1.5141	0.1165	0.8851
4	0.7585	0.0583	0.9434

**Table A10:** Eigenvectors (component loadings).

	PC 1	PC 2	PC 3	PC 4
Variable 1	−0.351	−0.063	−0.099	0.053
Variable 2	0.320	−0.195	0.198	−0.069
Variable 3	−0.045	0.560	−0.399	0.041
Variable 4	0.072	0.584	0.172	0.264
Variable 5	0.344	0.098	−0.137	0.065
Variable 6	0.327	0.005	−0.262	0.196
Variable7	−0.256	0.442	0.009	−0.128
Variable 8	−0.342	−0.138	−0.040	−0.155
Variable 9	0.317	−0.038	−0.274	−0.003
Variable 10	−0.215	−0.200	−0.329	0.663
Variable 11	0.164	0.054	0.576	0.532
Variable 12	−0.259	0.101	0.387	−0.031
GGE	0.306	0.156	0.094	−0.337

Note 1: GGE, “greenhouse gas emissions” (dependent variable). Source: own analysis.

### Appendix A.6. (Italy)

**Table A11:** Eigenanalysis of the correlation matrix.

Component	Eigenvalue	Proportion	Cumulative
**1**	8.8841	0.6834	0.6834
**2**	2.4143	0.1857	0.8691
**3**	0.9171	0.0705	0.9396
**4**	0.3005	0.0231	0.9628

**Table A12:** Eigenvectors (component loadings).

	PC 1	PC 2	PC 3	PC 4
Variable 1	−0.332	0.066	0.012	0.056
Variable 2	−0.313	−0.201	0.042	0.206
Variable 3	−0.160	0.423	0.506	0.502
Variable 4	−0.302	−0.084	0.220	−0.450
Variable 5	0.084	−0.604	−0.097	−0.073
Variable 6	0.024	−0.485	0.646	0.150
Variable7	−0.324	−0.018	−0.124	0.125
Variable 8	0.256	−0.277	−0.238	0.612
Variable 9	0.303	0.126	0.317	−0.243
Variable 10	0.315	−0.032	0.248	−0.024
Variable 11	−0.326	−0.068	−0.011	−0.046
Variable 12	0.294	0.265	−0.133	0.061
GGE	0.330	0.010	0.125	−0.131

Note 1: GGE, “greenhouse gas emissions” (dependent variable). Source: own analysis.

### Appendix A.7. (Bulgaria)

**Table A13:** Eigenanalysis of the correlation matrix.

Component	Eigenvalue	Proportion	Cumulative
**1**	6.5467	0.5036	0.5036
**2**	2.6340	0.2026	0.7062
**3**	1.9286	0.1484	0.8546
**4**	0.6976	0.0537	0.9082

**Table A14:** Eigenvectors (component loadings).

	PC 1	PC 2	PC 3	PC 4
Variable 1	−0.300	−0.266	0.036	0.294
Variable 2	0.336	0.186	0.054	−0.083
Variable 3	−0.247	0.427	0.014	0.234
Variable 4	−0.359	−0.059	0.195	−0.156
Variable 5	−0.320	−0.140	0.234	−0.099
Variable 6	−0.078	0.563	0.150	0.306
Variable7	−0.363	−0.026	−0.037	0.214
Variable 8	0.312	0.095	−0.313	0.377
Variable 9	−0.114	0.480	−0.277	−0.398
Variable 10	0.311	−0.296	0.133	−0.052
Variable 11	−0.142	−0.058	−0.589	−0.443
Variable 12	−0.363	−0.056	−0.068	−0.050
GGE	0.096	0.200	0.579	−0.420

Note 1: GGE, “greenhouse gas emissions” (dependent variable). Source: own analysis.

### Appendix A.8. (Czech Republic)

**Table A15:** Eigenanalysis of the correlation matrix.

Component	Eigenvalue	Proportion	Cumulative
**1**	7.2455	0.5573	0.5573
**2**	2.1307	0.1639	0.7212
**3**	1.6981	0.1306	0.8519
**4**	0.9810	0.0755	0.9273

**Table A16:** Eigenvectors (component loadings).

	PC 1	PC 2	PC 3	PC 4
Variable 1	−0.361	−0.094	−0.058	−0.093
Variable 2	0.310	0.307	−0.152	−0.153
Variable 3	−0.285	−0.235	0.115	0.184
Variable 4	−0.033	0.518	−0.436	0.227
Variable 5	0.320	−0.253	0.151	−0.206
Variable 6	0.322	−0.311	0.131	−0.020
Variable7	−0.318	0.027	−0.202	0.118
Variable 8	−0.310	−0.114	0.271	−0.210
Variable 9	0.264	−0.248	−0.426	0.152
Variable 10	0.226	−0.342	−0.300	0.315
Variable 11	−0.122	−0.023	−0.421	−0.758
Variable 12	−0.183	−0.461	−0.400	−0.070
GGE	0.344	0.090	0.085	−0.283

Note 1: GGE, “greenhouse gas emissions” (dependent variable). Source: own analysis.

### Appendix A.9. (Hungary)

**Table A17:** Eigenanalysis of the correlation matrix.

Component	Eigenvalue	Proportion	Cumulative
**1**	5.4146	0.4165	0.4165
**2**	3.1719	0.2440	0.6605
**3**	1.9573	0.1506	0.8111
**4**	1.4439	0.1111	0.9221

**Table A18:** Eigenvectors (component loadings).

	PC 1	PC 2	PC 3	PC 4
Variable 1	−0.348	−0.244	−0.109	−0.088
Variable 2	0.277	−0.128	0.347	0.360
Variable 3	−0.351	0.196	0.280	0.030
Variable 4	−0.407	−0.081	−0.059	−0.080
Variable 5	0.332	−0.175	−0.208	−0.324
Variable 6	0.386	0.094	0.018	−0.250
Variable7	−0.074	−0.389	0.034	0.473
Variable 8	0.377	−0.233	−0.098	−0.025
Variable 9	−0.033	0.315	−0.550	0.139
Variable 10	−0.021	0.397	−0.457	0.179
Variable 11	−0.177	−0.244	−0.029	−0.614
Variable 12	−0.130	0.398	0.395	−0.160
GGE	0.238	0.400	0.251	−0.106

Note 1: GGE, “greenhouse gas emissions” (dependent variable) Source: own analysis.

### Appendix A.10. (Poland)

**Table A19:** Eigenanalysis of the correlation matrix.

Component	Eigenvalue	Proportion	Cumulative
**1**	7.2837	0.5603	0.5603
**2**	2.9209	0.2247	0.7850
**3**	1.5369	0.1182	0.9032
**4**	0.7142	0.0549	0.9581

**Table A20:** Eigenvectors (component loadings).

	PC 1	PC 2	PC 3	PC 4
Variable 1	−0.369	−0.018	−0.010	−0.064
Variable 2	−0.174	−0.017	−0.610	0.043
Variable 3	−0.115	0.535	−0.080	−0.149
Variable 4	0.309	−0.237	−0.280	0.086
Variable 5	0.306	0.253	−0.269	0.050
Variable 6	0.327	0.152	−0.302	0.002
Variable7	−0.345	−0.093	−0.150	0.100
Variable 8	−0.279	0.182	−0.330	0.408
Variable 9	−0.275	−0.249	0.288	0.155
Variable 10	−0.362	0.119	−0.046	0.036
Variable 11	0.133	−0.475	−0.201	−0.424
Variable 12	−0.261	0.143	−0.127	−0.759
GGE	0.187	0.427	0.325	−0.070

Note 1: GGE, “greenhouse gas emissions” (dependent variable). Source: own analysis.

### Appendix A.11. (Romania)

**Table A21:** Eigenanalysis of the correlation matrix.

Component	Eigenvalue	Proportion	Cumulative
**1**	5.6896	0.4377	0.4377
**2**	2.8494	0.2192	0.6568
**3**	1.5549	0.1196	0.7765
**4**	1.1200	0.0862	0.8626

**Table A22:** Eigenvectors (component loadings).

	PC 1	PC 2	PC 3	PC 4
Variable 1	0.158	0.409	0.382	0.152
Variable 2	−0.332	−0.057	0.109	0.152
Variable 3	−0.306	0.371	0.060	0.154
Variable 4	−0.257	0.241	0.002	−0.511
Variable 5	0.392	0.167	−0.085	−0.054
Variable 6	0.259	0.131	−0.386	−0.264
Variable7	0.166	−0.143	0.551	−0.340
Variable 8	0.316	−0.319	−0.030	−0.302
Variable 9	0.322	0.041	0.238	0.229
Variable 10	0.216	−0.331	0.312	0.330
Variable 11	−0.277	−0.122	0.427	−0.415
Variable 12	0.092	0.517	0.204	0.044
GGE	0.348	0.270	0.004	−0.231

Note 1: GGE, “greenhouse gas emissions” (dependent variable). Source: own analysis.

### Appendix A.12. (Slovakia)

**Table A23:** Eigenanalysis of the correlation matrix.

Component	Eigenvalue	Proportion	Cumulative
**1**	7.3910	0.5685	0.5685
**2**	2.0843	0.1603	0.7289
**3**	1.5194	0.1169	0.8457
**4**	0.7455	0.0573	0.9031

**Table A24:** Eigenvectors (component loadings).

	PC 1	PC 2	PC 3	PC 4
Variable 1	−0.350	−0.074	0.139	−0.244
Variable 2	0.358	−0.087	0.120	−0.040
Variable 3	−0.214	0.481	−0.013	−0.244
Variable 4	−0.009	−0.573	−0.342	0.306
Variable 5	0.350	−0.096	−0.001	−0.003
Variable 6	0.296	0.270	0.229	−0.110
Variable7	−0.303	0.219	0.020	0.246
Variable 8	−0.132	0.358	−0.530	0.036
Variable 9	0.127	−0.149	−0.527	−0.696
Variable 10	−0.263	−0.287	0.185	−0.283
Variable 11	−0.233	−0.219	0.420	−0.290
Variable 12	−0.348	−0.094	−0.140	−0.067
GGE	0.352	0.086	0.092	−0.241

Note 1: GGE, “greenhouse gas emissions” (dependent variable). Source: own analysis.
